# Further Properties of Tsallis Entropy and Its Application

**DOI:** 10.3390/e25020199

**Published:** 2023-01-19

**Authors:** Ghadah Alomani, Mohamed Kayid

**Affiliations:** 1 Department of Mathematical Sciences, College of Science, Princess Nourah Bint Abdulrahman University, P.O. Box 84428, Riyadh 11671, Saudi Arabia; 2 Department of Statistics and Operations Research, College of Science, King Saud University, P.O. Box 2455, Riyadh 11451, Saudi Arabia

**Keywords:** coherent system, Shannon’s entropy, Tsallis entropy, system signature, stochastic orders

## Abstract

The entropy of Tsallis is a different measure of uncertainty for the Shannon entropy. The present work aims to study some additional properties of this measure and then initiate its connection with the usual stochastic order. Some other properties of the dynamical version of this measure are also investigated. It is well known that systems having greater lifetimes and small uncertainty are preferred systems and that the reliability of a system usually decreases as its uncertainty increases. Since Tsallis entropy measures uncertainty, the above remark leads us to study the Tsallis entropy of the lifetime of coherent systems and also the lifetime of mixed systems where the components have lifetimes which are independent and further, identically distributed (the iid case). Finally, we give some bounds on the Tsallis entropy of the systems and clarify their applicability.

## 1. Introduction

The Shannon entropy measure of a probability distribution has found critical applications in numerous areas described in Shannon’s seminal work [1]. Information theory provides an uncertainty measure associated with unpredictable phenomena. If *X* is a non-negative random variable (rv) with absolutely continuous cumulative distribution function (cdf) F(x) and probability density function (pdf) f(x), the Shannon entropy is represented as follows

(1) $$H\left(X\right)=H\left(f\right)=-\int_{0}^{\infty }f\left(x\right)\log f\left(x\right)dx,$$

provided the integral is finite. The entropy of Tsallis with order α generalizes the Shannon entropy, defined by (see [2])

(2) $$\begin{array}{ccc} H_{\alpha }\left(X\right) & = & H_{\alpha }\left(f\right)=\frac{1}{\bar{\alpha }}\left[\int_{0}^{\infty }f^{\alpha }\left(x\right)dx-1\right],\\ & = & \frac{1}{\bar{\alpha }}\left[E\left(f^{\alpha -1}\left(F^{-1}\left(U\right)\right)\right)-1\right], \end{array}$$

where $\bar{\alpha }=1-\alpha$ and for all $\alpha \in D_{\alpha }=\left(0,1\right)\cup \left(1,\infty \right),$ where E(·) means the expectation and $F^{-1}\left(u\right)$ for u∈[0,1], stands for the quantile function. Generally, Tsallis entropy can be negative, however, it can be nonnegative if a proper value of α is chosen. It is evident that $H\left(f\right)=\lim_{\alpha \to 1}H_{\alpha }\left(f\right)$ and hence it reduces to the Shannon entropy.

It is known that the Shannon entropy is additive. The entropy of Tsallis is, however, non-additive since

$$H_{\alpha }\left(X,Y\right)=H_{\alpha }\left(X\right)+H_{\alpha }\left(Y\right)+\bar{\alpha }H_{\alpha }\left(X\right)H_{\alpha }\left(Y\right).$$

Due to the flexibility of the Tsallis entropy compared to the Shannon entropy, the non-additive entropy measures find their justification in many areas of information theory, physics, chemistry, and technology.

Several properties and statistical aspects of the Tsallis entropy can be found in [3,4,5,6]. The Tsallis entropy for coherent systems and also mixed systems in the iid case is studied. A novel measure of uncertainty involving differential Shannon entropy and discriminant Kullback–Leibler quantity for comparing systems in terms of uncertainty in predicting lifetimes has been developed by [7,8,9]. A similar consequence on the subject has been argued in [10]. Furthermore, the Tsallis entropy properties of the order statistics in [11]. We aim here to continue the research within an analogous framework.

The rest of the article is thus planned as follows. In Section 2, we first study some essential properties of the Tsallis entropy of order α and then establish sufficient conditions for it to preserve the usual stochastic order. Section 3 examines various properties of the dynamical version in detail. Section 4 discusses the Tsallis entropy and its properties for coherent structures and mixed structures in the iid case. Finally, bounds on the Tsallis entropy of system lifetimes are also given.

The stochastic orders $\leq_{st},\leq_{hr}$ and $\leq_{d}$, known as usual stochastic order, hazard rate order and dispersive order will be utilized in the rest of the paper (see Shaked and Shanthikumar [12]).

The following implications hold:

$$\leq_{hr}⟹\leq_{st}\;\mathrm{and}\;\leq_{d}⟹\leq_{st}.$$

[The] dispersive order is recognized to be the order of distributional variability.

## 2. Properties of Tsallis Entropy

Below are other useful properties of the measure. First, another useful expression for the Tsallis entropy can be conveyed in terms of the proportional hazard rate (PH) function. For this purpose, if $X\in R^{+}$ is related to S(x), or the hazard rate function λ(x)=f(x)/S(x), the Tsallis entropy is expressed as

(3) $$H_{\alpha }\left(f\right)=\frac{1}{1-\alpha }\left[E(\lambda^{\alpha -1}\left(X_{\alpha }\right))-1\right],$$

for all $\alpha \in D_{\alpha },$ where $X_{\alpha }$ denotes the rv with pdf

(4) $$p_{\alpha }\left(x\right)=\alpha f\left(x\right)S^{\alpha -1}\left(x\right).$$

In Table 1, we provided the Tsallis entropy of some well-known distributions.

It is worth noting that the pdf given in (4), is actually the pdf of the minimum of two rv’s in the iid case (see, [13]). We note that the Tsallis entropy is invariant in the discrete case, while it is not invariant in the continuous case under one-to-one transformation of the rv under consideration. In this case, if ϕ(·):R↦R is a one-to-one function and $X_{2}=\phi \left(X_{1}\right),$ then (see e.g., Ebrahimi et al. [14])

$$H\left(X_{2}\right)=H\left(X_{1}\right)-E\left[\log J_{\phi }\left(X_{1}\right)\right],$$

where $J_{\phi }\left(X_{2}\right)=|\frac{d\phi^{-1}\left(X_{2}\right)}{dX_{2}}|$ is the Jacobian of the transformation. It is evident that $f_{X_{2}}\left(x\right)=f_{X_{1}}\left(\phi^{-1}\left(x\right)\right)\left|\frac{1}{\phi^{\prime }\left(\phi^{-1}\left(x\right)\right)}\right|.$ Hence, one can readily find that

$$\begin{array}{ccc} H_{\alpha }\left(X_{2}\right) & = & \frac{1}{1-\alpha }\left[\int_{0}^{\infty }f_{X_{2}}^{\alpha }\left(x\right)dx-1\right]\\ & = & \frac{1}{1-\alpha }\left[\int_{0}^{\infty }f_{X_{1}}^{\alpha }\left(\phi^{-1}\left(x\right)\right){\left(\frac{1}{|\phi^{\prime }\left(\phi^{-1}\left(x\right)\right)|}\right)}^{\alpha }dx-1\right]\\ & = & \frac{1}{1-\alpha }\left[\int_{0}^{\infty }\frac{f_{X_{1}}^{\alpha }\left(u\right)}{|\phi^{\prime }{\left(u\right)|}^{\alpha -1}}du-1\right], \end{array}$$

for all $\alpha \in D_{\alpha }.$ The Shannon entropy is scale-dependent. However, it is free of location, that is, *X* retains the identical differential entropy as X+b, for any b∈R. The same results also hold for the Tsallis entropy. Indeed, for all a≥0 and b∈R, from the above relation, we have

$$H_{\alpha }\left(aX+b\right)=a^{1-\alpha }H_{\alpha }\left(X\right)+\frac{1-a^{\alpha -1}}{1-\alpha }.$$

Now, we recall the definition of Tsallis entropy which can be seen in [4] for greater details.

**Definition 1.**  *Suppose $X_{1},X_{2}\in R^{+}$ with the cdfs $F_{1},F_{2}.$ The rv $X_{1}$ is smaller than the rv $X_{2}$ in the Tsallis entropy of order α, ($X_{1}\leq_{TE}X_{2}$) if $H_{\alpha }\left(X_{1}\right)\leq H_{\alpha }\left(X_{2}\right)$ for all $\alpha \in D_{\alpha }.$*

It is worth pointing out that $X_{1}\leq_{TE}X_{2}$ indicates that the predictability of the outcome of $X_{1}$ is more than that of $X_{2}$ in terms of the Tsallis entropy. As an immediate consequence of (2), consider the subsequent theorem.

**Theorem 1.**  *Let $X_{1},X_{2}\in R^{+}$ with cdfs $F_{1}$ and $F_{2}$, respectively. If $X_{1}\leq_{d}X_{2},$ then $X_{1}\leq_{TE}X_{2}.$*

**Proof.**  If $X_{1}\leq_{d}X_{2},$ then, for all α≥(≤)1

$$\left(1-\alpha \right)H_{\alpha }\left(X_{2}\right)=\int_{0}^{1}f_{2}^{\alpha -1}\left(F_{2}^{-1}\left(u\right)\right)du-1\geq \left(\leq \right)\int_{0}^{1}f_{1}^{\alpha -1}\left(F_{1}^{-1}\left(u\right)\right)du-1=\left(1-\alpha \right)H_{\alpha }\left(X_{1}\right).$$

This yields $H_{\alpha }\left(X_{1}\right)\leq H_{\alpha }\left(X_{2}\right)$, for all α>0. □

The following theorem develops the impact of a transformation on the Tsallis entropy of an rv. It is analogous to Theorem 1 in [15] and hence we skip its proof.

**Theorem 2.**  *Let $X_{1}\in R^{+}$ with the pdf $f_{1}\left(x\right)$ and $X_{2}=\phi \left(X_{1}\right)$, where ϕ:(0,∞)→(0,∞) is a function with a continuous derivative $\phi^{\prime }\left(x\right)$ such that $E(X_{2}^{2})<\infty .$ If $|\phi^{\prime }\left(x\right)|\geq 1$ for all x supported by $X_{1},$ then $H_{\alpha }\left(X_{1}\right)\leq H_{\alpha }\left(X_{2}\right)$ for all $\alpha \in D_{\alpha }.$*

The next theorem presents implications of the stochastic order under some aging constraints of the associated rv’s and the order α.

**Theorem 3.**  *If $X_{1}\leq_{st}X_{2},$ and*

**(i)**  *$X_{1}$ is DFR, then $H_{\alpha }\left(X_{1}\right)\leq H_{\alpha }\left(X_{2}\right)$ for all 0≤α≤1;*
**(ii)**  *$X_{2}$ is DFR, then $H_{\alpha }\left(X_{1}\right)\leq H_{\alpha }\left(X_{2}\right)$ for all α≥1.*

**Proof.**  (i) Let $X_{1}\leq_{st}X_{2}$ and 0≤α≤1. If $X_{1}$ is DFR, then

(5) $$\begin{array}{ccc} \int_{0}^{\infty }f_{1}^{\alpha }\left(x\right)dx & \leq & \int_{0}^{\infty }f_{2}\left(x\right)f_{1}^{\alpha -1}\left(x\right)dx\\ & \leq & {\left(\int_{0}^{\infty }f_{2}^{\alpha }\left(x\right)dx\right)}^{\frac{1}{\alpha }}{\left(\int_{0}^{\infty }f_{1}^{\alpha }\left(x\right)dx\right)}^{\frac{\alpha -1}{\alpha }}. \end{array}$$

The first inequality in (5) is obtained as follows. Since $X_{1}$ is DFR, then $f_{1}^{\alpha -1}\left(x\right)$ is increasing in *x* for all 0≤α≤1. So, the result ensues from the fact that $X_{1}\leq_{st}X_{2}$ implies $E\left[f_{1}^{\alpha -1}\left(X_{1}\right)\right]\leq E\left[f_{1}^{\alpha -1}\left(X_{2}\right)\right]$ for all increasing function $f_{1}^{\alpha -1}\left(x\right),\;x>0.$ The second inequality is obtained by using Hölder’s inequality. Thanks to the use of relations (2) and (5), we have

$$1+\left(1-\alpha \right)H_{\alpha }\left(X_{1}\right)\leq 1+\left(1-\alpha \right)H_{\alpha }\left(X_{2}\right),$$

which proves the claim. To prove (ii), we have

$$\begin{array}{ccc} \int_{0}^{\infty }f_{2}^{\alpha }\left(x\right)dx & \leq & \int_{0}^{\infty }f_{1}\left(x\right)f_{2}^{\alpha -1}\left(x\right)dx\\ & \leq & {\left(\int_{0}^{\infty }f_{1}^{\alpha }\left(x\right)dx\right)}^{\frac{1}{\alpha }}{\left(\int_{0}^{\infty }f_{2}^{\alpha }\left(x\right)dx\right)}^{\frac{\alpha -1}{\alpha }}. \end{array}$$

The first inequality is obtained by mentioning that $X_{2}$ is DFR and hence $f_{2}^{\alpha -1}\left(x\right)$ is decreasing in *x* for all α≥1 while the second inequality is given by using Hölder’s inequality. Now, the results follow. □

It is worth noting that Theorem 3 can be applied to several statistical models such as Weibull, Rayleigh, Pareto, Gamma, and Half Logistic, among others. The mentioned models involve the DFR aging property by choosing a suitable parameter.

## 3. Properties of Residual Tsallis Entropy

Here we give an overview of the dynamical perspectives of the Tsallis entropy of order α. We note that in this section, the term “decreasing” is equivalent to “non-increasing”, and “increasing” is equivalent to “non-decreasing”. Suppose that *X* is the life length of a new system. In this case, the Tsallis entropy $H_{\alpha }\left(X\right)$ is appropriate to measure the uncertainty of such a system. However, if the system remains alive until the age t, then $H_{\alpha }\left(X\right)$ is not appropriate to measure the remaining or residual uncertainty in the system’s lifetime. Thus, let us denote $X_{t}:=\left[X-t|X\geq t\right],$ the residual lifetime of an item with the pdf $f\left(x;t\right)=f\left(x\right)/S\left(t\right),\;x\in D_{t}$ where $D_{t}=\left\{x:x>t\right\}.$ Then, the residual Tsallis entropy is represented by

$$\begin{array}{c} H_{\alpha }\left(X;t\right)=\frac{1}{1-\alpha }\left[\int_{D_{t}}f^{\alpha }\left(x;t\right)dx-1\right]=\frac{1}{1-\alpha }\left[\int_{t}^{\infty }{\left[\frac{f(x)}{S(t)}\right]}^{\alpha }dx-1\right], \end{array}$$

for all t≥0 and $\alpha \in D_{\alpha }.$ Another useful representation is as follows:

(6) $$H_{\alpha }\left(X;t\right)=\frac{1}{1-\alpha }\left[\frac{E(\lambda^{\alpha -1}\left(X_{\alpha ,t}\right))}{\alpha }-1\right],$$

where $X_{\alpha ,t}$ denotes the rv having pdf

(7) $$p_{\alpha }\left(x;t\right)=\frac{\alpha f(x)}{S(t)}{\left(\frac{S(x)}{S(t)}\right)}^{\alpha -1},\;x\geq t>0.$$

Based on the measure $H_{\alpha }\left(X;t\right)$, a few classes of life distributions are proposed.

**Definition 2.**  *The rv X has increasing (decreasing) residual Tsallis entropy of order α ($IRTE_{\alpha }\left(DRTE_{\alpha }\right)$) if $H_{\alpha }\left(X;t\right)$ is increasing (decreasing) in t for all $\alpha \in D_{\alpha }.$*

Roughly speaking, if a unit has a cdf belonging to the class of $IRTE_{\alpha }\left(DRTE_{\alpha }\right)$, then the conditional pdf becomes less (more) informative as the unit becomes older. In the following lemma, we give the derivative of the residual Tsallis entropy.

**Lemma 1.**  *For all t≥0, we have*

(8) $$H_{\alpha }^{\prime }\left(X;t\right)=\frac{\lambda (t)}{1-\alpha }\left[E\left(\lambda^{\alpha -1}\left(X_{\alpha ,t}\right)\right)-\lambda^{\alpha -1}\left(t\right)\right],$$

*for all $\alpha \in D_{\alpha }.$*

**Remark 1.**  *Let us assume that X is $IRTE_{\alpha }\left(DRTE_{\alpha }\right).$ Then, $H_{\alpha }^{\prime }\left(X;t\right)=0$ and therefore we have $E\left(\lambda^{\alpha -1}\left(X_{\alpha ,t}\right)\right)=\lambda^{\alpha -1}\left(t\right).$ That is λ(t)=λ,t≥0. This reveals that exponential distribution is the only distribution fulfilling $IRTE_{\alpha }$ and $DRTE_{\alpha }.$*

We establishes a relationship among the new and known classes of life distributions with increasing (decreasing) failure rates.

**Theorem 4.**  *For any $X\in R^{+}$ with the pdf f, if X has IFR(DFR) property, then X is $DRTE_{\alpha }\left(IRTE_{\alpha }\right)$.*

**Proof.**  We shall prove it for IFR, while the DFR case can be derived similarly. Suppose *X* is IFR and 0≤α≤1. Then $\lambda^{\alpha -1}\left(x\right)$ is decreasing and hence we have

(9) $$E\left(\lambda^{\alpha -1}\left(X_{\alpha ,t}\right)\right)=\int_{t}^{\infty }\lambda^{\alpha -1}\left(x\right)p_{\alpha }\left(x;t\right)dx\leq \lambda^{\alpha -1}\left(t\right),$$

for t>0. Since 1−α≥0, from (8) the result follows. When α≥1, then the above relation is reversed and using again (8), we have the result and this implies that *X* is $DRTE_{\alpha }.$ □

There is a large class of monotonic density distributions to which the above theorem can be applied. Another important class of life distributions is those with an increasing failure rate in average (IFRA). In particular, *X* is IFRA if −logS(x)/x increases in x. The following example shows no relationship between the proposed class and the IFRA class of life distributions.

**Example 1.**  *Consider the sf of a random lifetime as*

$$S\left(x\right)=1-\left(1-e^{-0.2x}\right)\left(1-e^{-3x}\right),$$

*and hence*

$$f\left(x\right)=0.2e^{-0.2x}\left(1-e^{-3x}\right)+3\left(1-e^{-0.2x}\right)e^{-3x}.$$

**Figure 1:**
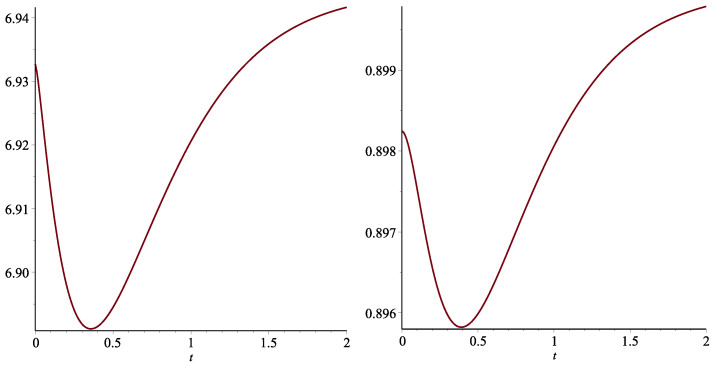
The residual Tsallis entropy of order α=0.5 (left panel) and α=2 (right panel) with respect to *t* given in Example 1.

*The plot of the residual Tsallis entropy in Figure 1 exhibits that X is not $DRTE_{\alpha }$.*

We now see how the residual Tsallis entropy and the hazard rate orders are related.

**Theorem 5.**  *If $X_{1}\leq_{hr}X_{2}$ and either $X_{1}$ or $X_{2}$ is DFR, then $H_{\alpha }\left(X_{1};t\right)\leq H_{\alpha }\left(X_{2};t\right)$ for all $\alpha \in D_{\alpha }.$*

**Proof.**  Suppose $X_{1,t}$ and $X_{2,t}$ denote the residual rv’s with pdfs $f_{1,t}$ and $f_{2,t},$ respectively. The relation $X_{1}\leq_{hr}X_{2}$ implies that $\left[X_{1,\alpha }-t|X_{1,\alpha }>t\right]\leq_{st}\left[X_{2,\alpha }-t|X_{2,\alpha }>t\right],$ where $X_{1,\alpha }$ and $X_{2,\alpha }$ have sf’s $S_{1}^{\alpha }\left(x\right)$ and $S_{2}^{\alpha }\left(x\right),$ respectively. Let us suppose α≥1. If we assume that $X_{1}$ is DFR, then $\lambda_{1}^{\alpha -1}\left(x\right)$ is decreasing in x. Hence, we have

$$E\left[\lambda_{1}^{\alpha -1}\left(X_{1,\alpha ,t}\right)\right]\geq E\left[\lambda_{2}^{\alpha -1}\left(X_{2,\alpha ,t}\right)\right]\geq E\left[\lambda_{2}\left(X_{1,\alpha ,t}\right)\right],$$

for all t≥0, where $X_{i,\alpha ,t}=\left[X_{i,\alpha }-t|X_{i,\alpha }>t\right],\;\left(i=1,2\right).$ From (6), we obtain $H_{\alpha }\left(X_{1};t\right)\leq H_{\alpha }\left(X_{2};t\right)$ for all α≥1. Now if we assume that 0≤α≤1, then the above relation can be reversed and then from (6), we obtain $H_{\alpha }\left(X_{1};t\right)\leq H_{\alpha }\left(X_{2};t\right)$ for all 0≤α≤1. So, we have $H_{\alpha }\left(X_{1};t\right)\leq H_{\alpha }\left(X_{2};t\right)$ for all $\alpha \in D_{\alpha }.$ The proof when $X_{2}$ is DFR can be obtained similarly. □

## 4. Tsallis Entropy of Coherent Structures and Mixed Structures

This section gives some aspects of the Tsallis entropy of coherent (and mixed) structures. A coherent system is one in which all system components are relevant, and the structure-function of it is monotonic. The *k*-out-of-*n* system is a particular case of a coherent structure. Furthermore, a mixture of coherent systems is considered as a mixed system (see, [16]). The reliability function of the mixed system lifetime *T*, in the iid case, is represented by

(10) $$\begin{array}{c} S_{T}\left(t\right)=\sum_{i=1}^{n}p_{i}S_{i:n}\left(t\right), \end{array}$$

where Si:n(t)=∑j=0i−1nj[F(t)]j[S(t)]n−j when i=1,⋯,n are the sf’s of $X_{1:n},\cdots ,X_{n:n}.$ The density function of *T* can be written as

(11) $$\begin{array}{c} f_{T}\left(t\right)=\sum_{i=1}^{n}p_{i}f_{i:n}\left(t\right), \end{array}$$

so that

(12) $$\begin{array}{c} f_{i:n}\left(t\right)=\frac{\Gamma (n+1)}{\Gamma (i)\Gamma (n-i+1)}{\left[F\left(t\right)\right]}^{i-1}{\left[S\left(t\right)\right]}^{n-i}f\left(t\right),\;1\leq i\leq n, \end{array}$$

The vector $p=(p_{1},\cdots ,p_{n})$ in $S_{T}\left(t\right)$ is called the *system signature*, such that $p_{i}=P\left(T=X_{i:n}\right).$ Note that $p_{1},\cdots ,p_{n}$ are non-negative as they are probabilities and thus $\sum_{i=1}^{n}p_{i}=1$ holds.

The order statistic $U_{i:n}=F\left(X_{i:n}\right)$ has pdf

(13) $$g_{i}\left(u\right)=\frac{\Gamma (n+1)}{\Gamma (i)\Gamma (n-i+1)}u^{i-1}{\left(1-u\right)}^{n-i}.$$

Now, the pdf of V=F(T) is given by

(14) $$g_{V}\left(v\right)=\sum_{i=1}^{n}p_{i}g_{i}\left(v\right).$$

Applying the above transformations, we find in the following theorem a formula for the Tsallis entropy of T.

**Proposition 1.**  *The Tsallis entropy of T is*

(15) $$H_{\alpha }\left(T\right)=\frac{1}{1-\alpha }\left[\int_{0}^{1}g_{V}^{\alpha }\left(v\right)f^{\alpha -1}\left(F^{-1}\left(v\right)\right)dv-1\right],$$

*where $g_{V}\left(v\right)$ has been shown in (14).*

**Proof.**  By using the change of v=F(x), from (2) and (11) we have

(16) $$\begin{array}{ccc} H_{\alpha }\left(T\right) & = & \frac{1}{1-\alpha }\left[E\int_{0}^{\infty }{\left(\sum_{i=1}^{n}p_{i}f_{i:n}\left(t\right)\right)}^{\alpha }dt-1\right],\\ & = & \frac{1}{1-\alpha }\left[\int_{0}^{1}{\left(\sum_{i=1}^{n}p_{i}\frac{\Gamma (n+1)}{\Gamma (i)\Gamma (n-i+1)}v^{i-1}{\left(1-v\right)}^{n-i}\right)}^{\alpha }f^{\alpha -1}\left(F^{-1}\left(v\right)\right)dv-1\right],\\ & = & \frac{1}{1-\alpha }\left[\int_{0}^{1}g_{V}^{\alpha }\left(v\right)f^{\alpha -1}\left(F^{-1}\left(v\right)\right)dv-1\right], \end{array}$$

for all $\alpha \in D_{\alpha }.$ The result now follows. □

To apply Proposition 1, consider the next example.

**Example 2.**  *The vector s=(0,0.2,0.6,0.2,0) is the signature of a bridge system with n=5 in the iid case with the basal reliability function S(t)=exp(−λt). This system remains functional provided that there is a path of operational connections running from left to right. It is obvious that $f(F^{-1}\left(v\right))=\lambda \left(1-v\right),\;0\leq v\leq 1,$ and we therefore have*

$$H_{\alpha }\left(T\right)=\frac{1}{1-\alpha }\left[\lambda^{\alpha -1}\int_{0}^{1}g_{V}^{\alpha }\left(v\right){\left(1-v\right)}^{\left(\alpha -1\right)}dv-1\right],$$

*for all $\alpha \in D_{\alpha }.$ The Tsallis entropy is decreasing with respect to λ as the uncertainty of the system’s lifetime decreases with increasing the parameter λ. Moreover, we have $H_{0.2}\left(T\right)=2.6952,H_{1.5}\left(T\right)=0.4670,H_{2}\left(T\right)=0.3682,H_{2.5}\left(T\right)=0.3062.$ It is clear that it decreases as α increases.*

The system signatures of orders 1–5 have been calculated in [17] and, therefore, we can compute the values of $H_{\alpha }\left(T\right)$ numerically for all α≥0. Considering different values of α, the Tsallis entropy of systems with 1–4 iid exponential components has been given in Table 2. Generally, $H_{\alpha }\left(T\right)$ has well resulted concerning the standard deviations of $T_{\alpha }$ for some α as was shown in Table 2. To compare the Tsallis entropy of two mixed systems with the identical signature having iid component lifetimes by using Equation (15) which is expressed below.

**Theorem 6.**  *The rv’s $T^{X_{1}}$ and $T^{X_{2}}$ are supposed to be the lifetime of two mixed systems with the same signature having n iid component lifetimes.*

*(i) If $X_{1}\leq_{d}X_{2}$, then $T^{X_{1}}\leq_{TE}T^{X_{2}}$.*

*(ii) Assume $X_{1}\leq_{TE}X_{2}$ and consider*

$$\begin{array}{c} B_{1}=\left\{0<v<1|\frac{f_{2}\left(F_{2}^{-1}\left(v\right)\right)}{f_{1}\left(F_{1}^{-1}\left(v\right)\right)}<1\right\},B_{2}=\left\{0<v<1|\frac{f_{2}\left(F_{2}^{-1}\left(v\right)\right)}{f_{1}\left(F_{1}^{-1}\left(v\right)\right)}\geq 1\right\}. \end{array}$$

*If $B_{1}=\phi$, $B_{2}=\phi$ or $\inf_{B_{1}}g_{V}\left(v\right)\geq \sup_{B_{2}}g_{V}\left(v\right)$, then $T^{X_{1}}\leq_{TE}T^{X_{2}}$.*

**Proof.**  (i) By the assumption $X\leq_{d}Y$ and the two systems have the same signature, so expression (15) gives

$$\left(1-\alpha \right)H_{\alpha }\left(T^{X_{1}}\right)-\left(1-\alpha \right)H_{\alpha }\left(T^{X_{2}}\right)=\int_{0}^{1}g_{V}^{\alpha }\left(v\right)\left(f_{1}^{\alpha -1}\left(F_{1}^{-1}\left(v\right)\right)-f_{2}^{\alpha -1}\left(F_{2}^{-1}\left(v\right)\right)\right)dv\leq 0,$$

for all 0<α<1 and

$$\left(1-\alpha \right)H_{\alpha }\left(T^{X_{1}}\right)-\left(1-\alpha \right)H_{\alpha }\left(T^{X_{2}}\right)=\int_{0}^{1}g_{V}^{\alpha }\left(v\right)\left(f_{1}^{\alpha -1}\left(F_{1}^{-1}\left(v\right)\right)-f_{2}^{\alpha -1}\left(F_{2}^{-1}\left(v\right)\right)\right)dv\geq 0,$$

for α>1. Now, this completes the proof.

(ii) If the condition $B_{1}=\phi$ or $B_{2}=\phi$ hold, then the outcome is clear. Hence, we assume that $B_{1}\neq \phi$ and $B_{2}\neq \phi$. Assumption $X\leq_{TE}Y$ and Equation (2) imply, for α>1,

(17) $$\int_{0}^{1}\left(f_{1}^{\alpha -1}\left(F_{1}^{-1}\left(v\right)\right)-f_{2}^{\alpha -1}\left(F_{2}^{-1}\left(v\right)\right)\right)dv\geq 0,$$

and for 0<α<1,

$$\int_{0}^{1}\left(f_{1}^{\alpha -1}\left(F_{1}^{-1}\left(v\right)\right)-f_{2}^{\alpha -1}\left(F_{2}^{-1}\left(v\right)\right)\right)dv\leq 0.$$

Let us assume

$$\left(1-\alpha \right)H_{\alpha }\left(T^{X_{1}}\right)-\left(1-\alpha \right)H_{\alpha }\left(T^{X_{2}}\right)=\int_{0}^{1}g_{V}^{\alpha }\left(v\right)\left(f_{1}^{\alpha -1}\left(F_{1}^{-1}\left(v\right)\right)-f_{2}^{\alpha -1}\left(F_{2}^{-1}\left(v\right)\right)\right)dv.$$

Then, for all α>1, it holds that

(18) $$\begin{array}{ccc} & & \int_{B_{1}}g_{V}^{\alpha }\left(v\right)\left(f_{1}^{\alpha -1}\left(F_{1}^{-1}\left(v\right)\right)-f_{2}^{\alpha -1}\left(F_{2}^{-1}\left(v\right)\right)\right)dv\\ & & +\int_{B_{2}}g_{V}^{\alpha }\left(v\right)\left(f_{1}^{\alpha -1}\left(F_{1}^{-1}\left(v\right)\right)-f_{2}^{\alpha -1}\left(F_{2}^{-1}\left(v\right)\right)\right)dv\\ & & \geq {\left(\underset{B_{1}}{\inf}g_{V}\left(v\right)\right)}^{\alpha }\int_{B_{1}}\left(f_{1}^{\alpha -1}\left(F_{1}^{-1}\left(v\right)\right)-f_{2}^{\alpha -1}\left(F_{2}^{-1}\left(v\right)\right)\right)dv\\ & & +{\left(\underset{B_{2}}{\sup}g_{V}\left(v\right)\right)}^{\alpha }\int_{B_{2}}\left(f_{1}^{\alpha -1}\left(F_{1}^{-1}\left(v\right)\right)-f_{2}^{\alpha -1}\left(F_{2}^{-1}\left(v\right)\right)\right)dv\\ & & \geq {\left(\underset{B_{2}}{\sup}g_{V}\left(v\right)\right)}^{\alpha }\int_{B_{1}}\left(f_{1}^{\alpha -1}\left(F_{1}^{-1}\left(v\right)\right)-f_{2}^{\alpha -1}\left(F_{2}^{-1}\left(v\right)\right)\right)dv\\ & & +{\left(\underset{B_{2}}{\sup}g_{V}\left(v\right)\right)}^{\alpha }\int_{B_{2}}\left(f_{1}^{\alpha -1}\left(F_{1}^{-1}\left(v\right)\right)-f_{2}^{\alpha -1}\left(F_{2}^{-1}\left(v\right)\right)\right)dv\\ & = & {\left(\underset{B_{2}}{\sup}g_{V}\left(v\right)\right)}^{\alpha }\int_{0}^{1}\left(f_{1}^{\alpha -1}\left(F_{1}^{-1}\left(v\right)\right)-f_{2}^{\alpha -1}\left(F_{2}^{-1}\left(v\right)\right)\right)dv\geq 0. \end{array}$$

In (18), the second inequality is given by the assumption $\inf_{B_{1}}g_{V}\left(v\right)\geq \sup_{B_{2}}g_{V}\left(v\right)$ while the last inequality is found by implementing (17). The result for 0<α<1 can also be lifted as in the proof above. □

Let us take the following example to demonstrate the above theorem.

**Example 3.**  *Consider the lifetime $T^{X}=\min\left\{X_{1},\max\left\{X_{2},X_{3}\right\}\right\}$ in the iid case with basal cdf*

(19) $$F_{X}\left(t\right)=1-e^{-2t},$$

*and $T^{Z}=\min\left\{Z_{1},\max\left\{Z_{2},Z_{3}\right\}\right\}$ another lifetime with basal cdf*

(20) $$F_{Z}\left(t\right)=1-e^{-t}.$$

*The signature is $s=(\frac{1}{3},\frac{2}{3},0)$. It can be readily seen, for all $\alpha \in D_{\alpha },$ that*

$$H_{\alpha }\left(X\right)=\frac{2^{\alpha -1}-\alpha }{\alpha (1-\alpha )}\;\mathrm{and}\;H_{\alpha }\left(Z\right)=\frac{1}{\alpha }.$$

*So, one can see that $X\leq_{TE}Z$. Moreover, it is readily apparent that $B_{1}=\left[0,1\right)$ and $B_{2}=\left\{1\right\}$, and hence $\inf_{B_{1}}g_{V}\left(v\right)=\sup_{B_{2}}g_{V}\left(v\right)=0$. Therefore Part (ii) of Theorem 6 results $T^{X}\leq_{TE}T^{Z}$.*

We show that the minimum of lifetimes in the iid case has lower or equal Tsallis entropy than all mixed systems under the property of decreasing failure rate of component lifetimes.

**Theorem 7.**  *If T represents the mixed system’s lifetime in the iid case and the component lifetime is DFR, then $X_{1:n}\leq_{TE}T$.*

**Proof.**  Recalling [18], it is evident that $X_{1:n}\leq_{hr}T$ and $X_{1:n}\leq_{d}T$ as the series system has a DFR lifetime provided that the parent component lifetime is DFR. Now, the result is found by using Theorem 1. □

### Bounds for the Tsallis Entropy of Mixed Systems

With the insights from the previous section, we can now find some inequalities and bounds on the Tsallis entropy of mixed systems. We note that it is usually difficult or sometimes impossible to determine the Tsallis entropy when the system is identified by a complex structure-function or when the components included in the system are immeasurable. This is why it is valuable to provide bounds on the measure. The following result provides bounds for the Tsallis entropy in the mixed system concerning the Tsallis entropy of its components.

**Theorem 8.**  *If $H_{\alpha }\left(X\right)<\infty$. Then*

(21) $$H_{\alpha }\left(T\right)\geq {\left(\underset{v\in (0,1)}{\sup}g_{V}\left(v\right)\right)}^{\alpha }H_{\alpha }\left(X\right)+\frac{{\left(\sup_{v\in (0,1)}g_{V}\left(v\right)\right)}^{\alpha }-1}{1-\alpha },$$

*for all α>1 and*

(22) $$H_{\alpha }\left(T\right)\leq {\left(\underset{v\in (0,1)}{\sup}g_{V}\left(v\right)\right)}^{\alpha }H_{\alpha }\left(X\right)+\frac{{\left(\sup_{v\in (0,1)}g_{V}\left(v\right)\right)}^{\alpha }-1}{1-\alpha },$$

*for 0<α<1.*

**Proof.**  Recall the relation (15), for all $\alpha \in D_{\alpha },$ we have

$$\begin{array}{ccc} 1+\left(1-\alpha \right)H_{\alpha }\left(T\right) & = & \int_{0}^{1}g_{V}^{\alpha }\left(v\right)f^{\alpha -1}\left(F^{-1}\left(v\right)\right)dv\\ & & \leq {\left(\underset{v\in (0,1)}{\sup}g_{V}\left(v\right)\right)}^{\alpha }\int_{0}^{1}f^{\alpha -1}\left(F^{-1}\left(v\right)\right)dv\\ & = & {\left(\underset{v\in (0,1)}{\sup}g_{V}\left(v\right)\right)}^{\alpha }\left[\left(1-\alpha \right)H_{\alpha }\left(X\right)+1\right], \end{array}$$

where the last equality is obtained from (2) and this completes the proof. □

If the amount of the components of a system is very big or the system has a very complicated design, the provided bounds in (21) and (22) are very applicable in such circumstances. Here, we find an overall lower bound Tsallis entropy for the lifetime of the system by applying properties of Tsallis entropy.

**Theorem 9.**  *Underneath the requirements of the Theorem 8, we have*

(23) $$H_{\alpha }\left(T\right)\geq H_{\alpha }^{L}\left(T\right),$$

*where $H_{\alpha }^{L}\left(T\right)=\sum_{i=1}^{n}p_{i}H_{\alpha }\left(X_{i:n}\right)$ for all $\alpha \in D_{\alpha }$ and $H_{\alpha }\left(X_{i:n}\right)$ is the Tsallis entropy of the i-th order statistics.*

**Proof.**  Recall the Jensen’s inequality for the convex function $t^{\alpha }$ (it is concave (convex) for 0<α<1(α>1)), it holds that

$${\left(\sum_{i=1}^{n}p_{i}f_{i:n}\right)}^{\alpha }\geq \;\left(\leq \right)\sum_{i=1}^{n}p_{i}f_{i:n}^{\alpha }\left(t\right),\;t>0,$$

and hence we obtain

(24) $$\left(\int_{0}^{\infty }f_{T}^{\alpha }\left(t\right)dt\right)\geq \;\left(\leq \right)\left(\sum_{i=1}^{n}p_{i}\int_{0}^{\infty }f_{i:n}^{\alpha }\left(t\right)dt\right).$$

Since 1−α>0(1−α<0), by multiplying both side (24) in 1/(1−α), we obtain

$$\begin{array}{ccc} H_{\alpha }\left(T\right) & \geq & \frac{1}{1-\alpha }\left[\sum_{i=1}^{n}p_{i}\int_{0}^{\infty }f_{i:n}^{\alpha }\left(t\right)dt-1\right]\\ & = & \frac{1}{1-\alpha }\left[\sum_{i=1}^{n}p_{i}\int_{0}^{\infty }f_{i:n}^{\alpha }\left(t\right)dt-\sum_{i=1}^{n}p_{i}\right]\\ & = & \sum_{i=1}^{n}p_{i}\left[\frac{1}{1-\alpha }\left(\int_{0}^{\infty }f_{i:n}^{\alpha }\left(t\right)dt-1\right)\right]\\ & = & \sum_{i=1}^{n}p_{i}H_{\alpha }\left(X_{i:n}\right), \end{array}$$

and this completes the proof. □

The bound in (23) parallels the pdf of the lifetime of the system. It can be expressed as the linear transformation of the Tsallis entropy of an *i*-out-of-*n* system. Moreover, when the lower bound in Theorem 8 and also the lower bound in Theorem 9 can be determined, one may adopt the maximum of the two lower bounds.

**Example 4.**  *Let us suppose a system signature $s=(\frac{1}{3},\frac{2}{3},0)$ with lifetime T including n=3 iid component lifetimes having the common cdf given in (20). One can compute that $\sup_{x\in (0,1)}g_{V}\left(v\right)=1.333$ and hence recalling Theorem 8, the Tsallis entropy of T is bounded as follows:*

(25) $$H_{\alpha }\left(T\right)\geq \left(\leq \right){\left(1.333\right)}^{\alpha }H_{\alpha }\left(X\right)+\frac{{\left(1.333\right)}^{\alpha }-1}{1-\alpha },$$

**Figure 2:**
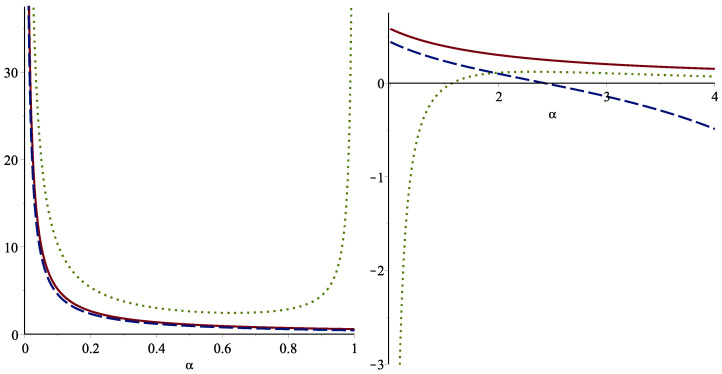
The bound in (25) (dotted line) and the lower bound given in (23) (dashed line) along with the exact value of $H_{\alpha }\left(T\right)$ for the standard exponential distribution concerning order α.

*for α>1(0<α<1). The component lifetimes are considered to be exponential. In this case, we plotted the bounds (25), (23) and the true value of $H_{\alpha }\left(T\right)$ using relation (15) which are shown in Figure 2. As it can be seen that the lower bound in (25) (dotted line) outperforms than (23) for α>2.*

## 5. Conclusions

Extensions of Shannon entropy have been presented in the literature before. The Tsallis entropy, which can be considered as a different measure of uncertainty, is one of the extended entropy measures. We have described here some other properties of this measure. We first determined the Tsallis entropy of some known distributions and then proved that it is not invariant if a one-to-one transformation of the lifetime is taken in the continuous case. The connection to the usual stochastic order has been revealed. It is well known that systems having greater lifetimes and lower uncertainty are apposite systems, and that the reliability of a system usually decreases as its uncertainty increases. These findings led us to study the entropy of Tsallis for coherent structures and mixed systems in the iid case. Finally, we established some bounds on the Tsallis entropy of the systems and illustrated the usefulness of the given bounds.

## Figures and Tables

**Table 1:** Tsallis entropy for some prominent distributions.

Distribution	f(x)	$H_{\alpha }\left(f\right)$
Uniform	$\frac{1}{\beta },\;0<x<\beta$	$\frac{b^{-\alpha }-1}{1-\alpha },\;b>0$
Gamma	$\frac{\lambda }{\Gamma (k)}x^{k-1}e^{-\lambda x},\;x>0$	$\frac{1}{1-\alpha }\left[\frac{\lambda^{\alpha -1}\Gamma \left(\alpha \left(k-1\right)+1\right)}{{\left(\alpha \lambda \right)}^{\alpha (k-1)}\Gamma^{\alpha }\left(k\right)}-1\right],\;k>0$
Weibull	$\frac{k}{\lambda }{\left(\frac{x}{\lambda }\right)}^{k-1}e^{-{\left(x/\lambda \right)}^{k}},\;x>0$	$\frac{1}{1-\alpha }\left[{\left(\frac{k}{\lambda }\right)}^{\alpha -1}\frac{\Gamma (\alpha (k-1)-k+2)}{\alpha^{\alpha (k-1)-k+2}}-1\right],\;k>0$
Beta	$\frac{1}{B(a,b)}x^{a-1}{\left(1-x\right)}^{b-1},\;0<x<1$	$\frac{1}{1-\alpha }\left[\frac{B(\alpha (a-1)+1,\alpha (b-1)+1)}{B^{\alpha }\left(a,b\right)}-1\right],\;a,b>0$

*B*(*a*, *b*) = Γ(*a* + *b*)/Γ(*a*)Γ(*b*) means the beta function and Γ(·) is the gamma function.

**Table 2:** Comparisons of Tsallis entropy and the lower bound of $T_{\alpha }$ for some values of α.

N	*p*	$H_{0.5}\left(T\right)$	$H_{1.5}\left(T\right)$	$H_{3}\left(T\right)$	$H_{0.5}^{L}\left(T\right)$	$H_{1.5}^{L}\left(T\right)$	$H_{3}^{L}\left(T\right)$
1	(1)	2.0000	0.6666	0.4000	2.0000	0.6666	0.4000
2	(1,0)	0.8284	0.1143	−0.1666	0.8284	0.1143	−0.1666
3	(0,1)	2.4428	0.8892	0.4333	2.4428	0.8892	0.4333
4	(1,0,0)	0.3094	−0.3094	−1.0000	0.3094	−0.3094	−1.0000
5	(1/3,2/3,0)	1.1111	0.3948	0.2023	1.9047	0.6309	0.2333
6	(0,1,0)	1.2659	0.5069	0.2857	1.2659	0.5069	0.2857
7	(0,2/3,1/3)	2.0698	0.7412	0.3845	1.7169	0.6527	0.3392
8	(0,0,1)	2.6188	0.9442	0.4464	2.6188	0.9442	0.4464
9	(1,0,0,0)	0.0000	−2.1666	−2.1666	0.0000	−2.1666	−2.1666
10	(1/2,1/2,0,0)	0.5030	−0.0375	−0.3242	0.3603	−0.2260	−1.0515
11	(1/4,3/4,0,0)	0.6407	0.1431	−0.0121	0.5405	−0.0057	−0.4939
12	(1/4,7/12,1/6,0)	0.9281	0.2788	0.1081	0.6644	0.0608	−0.4471
13	(1/4,1/4,1/2,0)	1.2514	0.4917	0.2732	0.9122	0.1941	−0.3536
14	(0,1,0,0)	0.7206	0.2145	0.0636	0.7206	0.2145	0.0636
15	(0,5/6,1/6,0)	0.9768	0.3318	0.1601	0.8445	0.2811	0.1103
16,17	(0,2/3,1/3,0)	1.1415	0.4292	0.2333	0.9684	0.3478	0.1571
18,19	(0,1/2,1/2,0)	1.2659	0.5069	0.2857	1.0924	0.4144	0.2038
20,21	(0,1/3,2/3,0)	1.3612	0.5646	0.3199	1.2163	0.4810	0.2506
22	(0,1/6,5/6,0)	1.4299	0.6012	0.3385	1.3402	0.5477	0.2974
23	(0,0,1,0)	1.4641	0.6143	0.3441	1.4641	0.6143	0.3441
24	(0,1/2,1/4,1/4)	2.0179	0.6950	0.3593	1.4044	0.5031	0.2307
25	(0,1/6,7/12,1/4)	2.1010	0.7655	0.3958	1.6522	0.6364	0.3242
26	(0,0,3/4,1/4)	2.0979	0.7653	0.3959	1.7761	0.7030	0.3709
27	(0,0,1/2,1/2)	2.4161	0.8751	0.4290	2.0882	0.7917	0.3978
28	(0,0,0,1)	2.7123	0.9691	0.4515	2.7123	0.9691	0.4515

## Data Availability

No new data were created or analyzed in this study. Data sharing is not applicable to this article.
